# Islet Remodeling in Female Mice with Spontaneous Autoimmune and Streptozotocin-Induced Diabetes

**DOI:** 10.1371/journal.pone.0102843

**Published:** 2014-08-07

**Authors:** Annette Plesner, Joris T. ten Holder, C. Bruce Verchere

**Affiliations:** 1 Departments of Pathology and Laboratory Medicine, Child & Family Research Institute, University of British Columbia, Vancouver, British Columbia, Canada; 2 Department of Surgery, Child & Family Research Institute, University of British Columbia, Vancouver, British Columbia, Canada; Children’s Hospital Boston/Harvard Medical School, United States of America

## Abstract

Islet alpha- and delta-cells are spared autoimmune destruction directed at beta-cells in type 1 diabetes resulting in an apparent increase of non-beta endocrine cells in the islet core. We determined how islet remodeling in autoimmune diabetes compares to streptozotocin (STZ)-induced diabetes. Islet cell mass, proliferation, and immune cell infiltration in pancreas sections from diabetic NOD mice and mice with STZ-induced diabetes was assessed using quantitative image analysis. Serial sections were stained for various beta-cell markers and Ngn3, typically restricted to embryonic tissue, was only upregulated in diabetic NOD mouse islets. Serum levels of insulin, glucagon and GLP-1 were measured to compare hormone levels with respect to disease state. Total pancreatic alpha-cell mass did not change as autoimmune diabetes developed in NOD mice despite the proportion of islet area comprised of alpha- and delta-cells increased. By contrast, alpha- and delta-cell mass was increased in mice with STZ-induced diabetes. Serum levels of glucagon reflected these changes in alpha-cell mass: glucagon levels remained constant in NOD mice over time but increased significantly in STZ-induced diabetes. Increased serum GLP-1 levels were found in both models of diabetes, likely due to alpha-cell expression of prohormone convertase 1/3. Alpha- or delta-cell mass in STZ-diabetic mice did not normalize by replacement of insulin via osmotic mini-pumps or islet transplantation. Hence, the inflammatory milieu in NOD mouse islets may restrict alpha-cell expansion highlighting important differences between these two diabetes models and raising the possibility that increased alpha-cell mass might contribute to the hyperglycemia observed in the STZ model.

## Introduction

Type 1 diabetes is caused by selective autoimmune destruction of the insulin-producing beta-cells of the pancreas [1], [2], [3]. The immune system solely targets the beta-cells, leaving other islet endocrine cell types including the glucagon-producing alpha-cells, the somatostatin-producing delta-cells and the pancreatic polypeptide-producing (PP) cells intact. In fact, increased proportions of both alpha and delta-cells have been reported in the pancreas of type 1 diabetic patients [4], in the non-obese diabetic (NOD) mouse model of type 1 diabetes [5], and in streptozotocin (STZ)-induced diabetes in rats [4], although a recent report suggested that alpha-cell mass declines in autoimmune diabetes [6]. Expansion of the alpha-cell population has also been reported in mice with diabetes induced by multiple low-doses of STZ [7]. The stimulus driving non-beta endocrine cell reorganization during the development of diabetes and the physiological significance of this phenomenon is unknown. However, a recent study in metabolically stressed mice with a beta-cell specific somatic mutation of the insulin regulatory gene FoxO1 has shown that dedifferentiated beta-cells progress to upregulate Ngn3, Oct4 and other beta-cell progenitor markers in addition to converting to expression of glucagon, somatostatin or PP [8]. As such, non-beta endocrine cells have been proposed to be progenitors capable of replenishing lost beta-cells [9], [10], [11], although other evidence suggests that replication of existing beta-cells [12], [13] or differentiation of non-endocrine pancreatic progenitors [14], [15], [16] are also important sources of new beta-cells, at least in adult mice. In addition, alpha-cell hyperplasia has been suggested to contribute to diabetic hyperglycemia through production of excess glucagon [17].

In the present study, we sought to assess the changes that occur in islet endocrine cell populations and identify factors that may be involved in driving these changes during development of autoimmune diabetes in the NOD mouse model of spontaneous autoimmune diabetes [18]. We compared NOD mice to animals with STZ-induced diabetes to determine whether the observed remodeling of non-beta islet endocrine cells is driven by increasing blood glucose or whether infiltrating immune cells present in the NOD model may stimulate or restrict islet cell proliferation. To address the significance of hyperglycemia in islet remodeling in diabetes we restored normoglycemia in STZ-diabetic animals by islet transplantation or implantation of an insulin mini-pump. Taken together our data indicate that multiple mechanisms are essential for non-beta islet endocrine cell remodeling in diabetic NOD mice and that these cells simply may redistribute to fill the void left by loss of beta-cells within the diabetic islet once insulitis dissipates.

## Materials and Methods

### Animals

Neonatal to 24-wk old female NOD mice (H*-*2^g7^, Taconics, Germantown, USA) and neonatal to 20-wk old female Balb/c mice (H-2^d^, Jackson, Bar Harbor, USA) were housed in the animal facility of the Child & Family Research Institute and maintained in strict accordance with the principles and guidelines of the Canadian Council on Animal Care including 12 h light/12 h dark cycle, group housing and water and chow ad libitum (Purina 5053 PicoLab Rodent Diet 20, LabDiet, St. Louis, USA). All studies were approved by the Animal Care Committee of the University of British Columbia and all surgery and euthanasia was performed using isoflurane or avertin to ensure minimal suffering (Animal Permit Numbers A06-1452 and A10-1085). Tail vein blood was collected from NOD mice weekly to monitor glucose levels from 4 wks of age using a glucometer (LifeScan, Burnaby, Canada). Every two weeks, 3–9 age-matched mice were euthanized for histological analysis of the pancreas, regardless of their blood glucose concentrations (Table 1). Mice were only included in the diabetic NOD mouse cohort if they exhibited sustained hyperglycemia for two weeks.

**Table 1:** Body weight and blood glucose levels of non-obese diabetic (NOD) mice studied.

Weeks	Weight (g)	Blood Glucose (mM)
4	14.4±0.6	8.6, 6.8, 8.2, 7.9, 6.8, 8.0, 7.6, 6.7, 7.4
6	18.1±0.6	6.8, 6.6, 7.6
8	20.9±0.6	6.6, 5.5, 6.6
10	21.4±1.4	6.6, 7.5, 5.6
12	22.2±0.3	5.3, 5.4, 5.4, 6.7, 4.9, 5.4, 7.2, 23.4
14	22.3±0.6	5.2, 6.2, 6.3
16	24.4±0.4	29.6, 7.8, 12.4, 7.2, 7.9, 6.5, 33.3, 32.7, 32.5
18	24.0±0.3	24.1, 29.6, 10.1
20	25.8±0.7	24.6, 6.2, 7.1
22	24.0±0.4	24.6, 23.3, 33.1, 33.2, 27.6
24	25.4±0.4	22.1, 33.1, 8.5, 18.1

Female NOD mice were analyzed every two weeks from age 4–24 weeks (n = 3–9 per group). Weights are presented as mean+/−SEM for each age group. Due to variability in onset of hyperglycemia blood glucose levels are listed as individual values.

To assess degree of endocrine cell proliferation 10 µl/g body weight of Bromodeoxyuridine (BrdU; Roche, Indianapolis, USA) in PBS was administered intravenously to groups of 4, 12 and 20 wk old NOD and Balb/c mice 24 h prior to euthanizing the animals.

Diabetes was induced in 10-wk old female Balb/c mice by a single intraperitoneal dose of 275 mg/kg STZ (Sigma, St Louis, USA). Within 24 h of induction of hyperglycemia (blood glucose ≥20 mM), some diabetic mice received an insulin implant (LinShin Canada, Scarborough, Canada), or transplantation of 300 syngeneic islets [19] to normalize blood glucose levels. For islet transplantation studies, islets from 10–12 wk old Balb/c mice were isolated by ductal collagenase injection, oscillating digestion, and purification on a dextran gradient. Islets were incubated overnight in complete Ham’s F-10 media (Gibco, Burlington, Canada), hand-counted into aliquots of 300 islets at least 100 µm in diameter, and transplanted into the left renal subcapsular space of age-matched hyperglycemic Balb/c mice under isoflourane anesthesia. Recipient mice were transplanted 3–5 days following STZ injection, when blood glucose levels were 22–26 mM on two consecutive days.

### Antibodies

Primary antibodies used for immunohistochemistry in this study included guinea pig anti-human insulin (1∶100; Dako, Glostrup, Denmark), rabbit anti-human glucagon (1∶75; Dako), mouse anti-somatostatin (1∶500; gift from Dr. C. McIntosh, Vancouver, Canada), guinea pig anti-rat pancreatic polypeptide (1∶1000; Linco, St. Charles, USA), rabbit anti-mouse Glut-2 (1∶500; Millipore, Temacula, USA), mouse anti-GLP1 (active, amidated C-terminal specific antibody; 1∶500; BioPorto, Gentofte, Denmark), rabbit anti-mouse Ngn3 (1∶500; gift from Drs. F. Lynn and M. German), rabbit anti-mouse PC1/3 (1∶500; gift from Dr. L. Devi, NY, USA), rabbit anti-mouse PDX-1 (1∶500; Chemicon, Temecula, USA), rat anti-mouse Ki-67 (1∶100; Dako), rat-anti mouse BrdU (1∶300; Accurate Chemicals, Westbury, USA) and rat anti-mouse CD45 (1∶25; BD Pharmingen, San Diego, USA).

Secondary antibodies were Texas Red donkey anti-rabbit or goat anti-guinea pig, (both 1∶100, from Jackson Immuno Research, West Grove, USA); Alexa 594 goat anti-rat (1∶200) or Alexa 488 goat anti-mouse, goat anti-rat, goat anti-rabbit, or goat anti-guinea pig (all 1∶100; Molecular Probes, Eugene, USA). All antibodies were diluted in phosphate buffer saline (PBS) with 1% BSA or in blocking buffer (PBS with 2–5% serum).

### Immunohistochemistry

Pancreata were fixed in 4% paraformaldehyde in PBS (pH 7.5) for 1 h and stored at 4°C in 70% ethanol until embedded in paraffin. Five micrometer sections were deparaffinized in xylene, rehydrated in graded ethanol and distilled water and washed in PBS. Sections were incubated for 30–60 minutes with a blocking buffer containing 2–5% v/v serum followed by incubation with primary antibodies for 1 h at room temperature or overnight at 4° C, washed and incubated with the respective secondary antibodies for a further 1 h at room temperature. Antigen retrieval was necessary prior to immunostaining for BrdU, GLP1, Ki-67, PC1/3 and PDX-1 (10 mM citrate buffer; pH 6.0), and CD45 (Target Retrieval Solution; Dako).

### Insulitis and Glucagon Score

The severity of insulitis was assessed according to the following score: 0 =  normal islet with no or minimal, diffuse infiltration or islet where infiltration has dissipated (diabetic group only), 1 =  less than 1/3 of the islet infiltrated, 2 =  between 1/3 and 1/2 of the islet infiltrated, 3 =  more than 1/2 of the islet infiltrated and 4 =  islets with full insulitis [20]. The degree of glucagon-positive cells per islet area was assessed according to the following score: 0 =  islet with no alpha-cells, 1 =  normal islet with peripheral alpha-cell location, 2 =  less than 1/3 of the islet comprised of alpha-cells, 3 =  between 1/3 and 1/2 of the islet comprised of alpha-cells and 4 =  more than 1/2 of the islet comprised of alpha-cells. All islets within each pancreas section were scored, with a minimum of 12 islets per section.

### Quantitative Image Analysis

To quantify alpha-, beta- and delta-cell area in NOD and Balb/c mouse islets and pancreata, we compared mice at 4 wks (n = 4–6), 12 wks (n = 4–6) and 18–24 wks (n = 4–6) of age. The young (4-week old) NOD group was euglycemic and pre-insulitic, the 12 week old NOD group was euglycemic and insulitic and the 18–24 week old NOD group was hyperglycemic and insulitic. Three 5 µm sections of each pancreas were cut 200 µm apart and immunostained for insulin, glucagon, and somatostatin. Sections used for quantification were assigned a random number to blind the observer. All visible islets on each section were captured and scored (mean number of islets analyzed per mouse was 24.1±1.3). The total number of islets analyzed in the 4 wk old group was 86 (n = 3) for NOD and 324 (n = 5) for Balb/c, 100 (n = 3) for NOD and 229 (n = 5) for Balb/c in the 12 wk old group and 133 (n = 5) for NOD and 316 (n = 5) for Balb/c in the 20 wk old. Islets were captured with a Zeiss Axioplan 2 fluorescent microscope and the area (mm^2^) that each different cell type occupied within each islet was calculated as a proportion of total islet area (mm^2^) using IPlab v.3.2 Imaging Software. Whole pancreas sections were captured using a BX61 microscope (Olympus, Tokyo, Japan) with QED acquisition software (Media Cybernetics, Silver Spring, USA), and the area (mm^2^) calculated using ImagePro Plus analysis software (Media Cybernetics). The percentage of pancreas area comprised of alpha-, beta- and delta-cells was calculated by dividing the respective endocrine cell area by the total area of the section and multiplying by 100. Endocrine cell mass (mg) was calculated by multiplying the percent endocrine cell area by the weight of the pancreas.

Three serial sections per mouse 200 µm apart (similar to quantifying endocrine cell mass) were immunostained for BrdU and glucagon, insulin or somatostatin and 10 islets per serial section (30 islets per mouse) were captured. Endocrine cell proliferation was calculated as the number of BrdU-positive endocrine cells (glucagon, insulin or somatostatin) divided by the total cell count of each respective endocrine cell type, and expressed as percent BrdU-positive cell type/day.

### Luminex-based assessment of hormones in mouse serum

Serum from female NOD mice (4, 12 and 20 wk) and 12-wk old Balb/c mice with or without STZ-induced diabetes was collected and DPP-IV inhibitor (Millipore, St. Charles, MI, USA) was added immediately to prevent degradation of GLP-1. Levels of insulin, glucagon and active GLP-1 were assessed using the Milliplex Map Kit according to the manufacturer’s instructions (Millipore, Billerica, MA, USA). Assays were read using Luminex xMAP Technology on a CS1000 Autoplex Analyzer (Perkin Elmer) and analyzed using MasterPlex QT software.

### Statistical analysis

Endocrine cell area is expressed as mean ± S.E.M. The data were compared using Student’s t-test or one-way ANOVA followed by Tukey’s Multiple Comparison Test. Correlation of alpha and delta-cell area, and of glucagon and insulitis score among individual islets was assessed using linear regression analysis. All statistical analyses were performed using Prism (GraphPad Software, Inc, San Diego, CA, USA). *p*<0.05 was considered significant.

## Results

### Similar Alpha-cell Mass in Pre-diabetic NOD Mice and Diabetic Mice with Sustained Hyperglycemia

**Figure 1:**
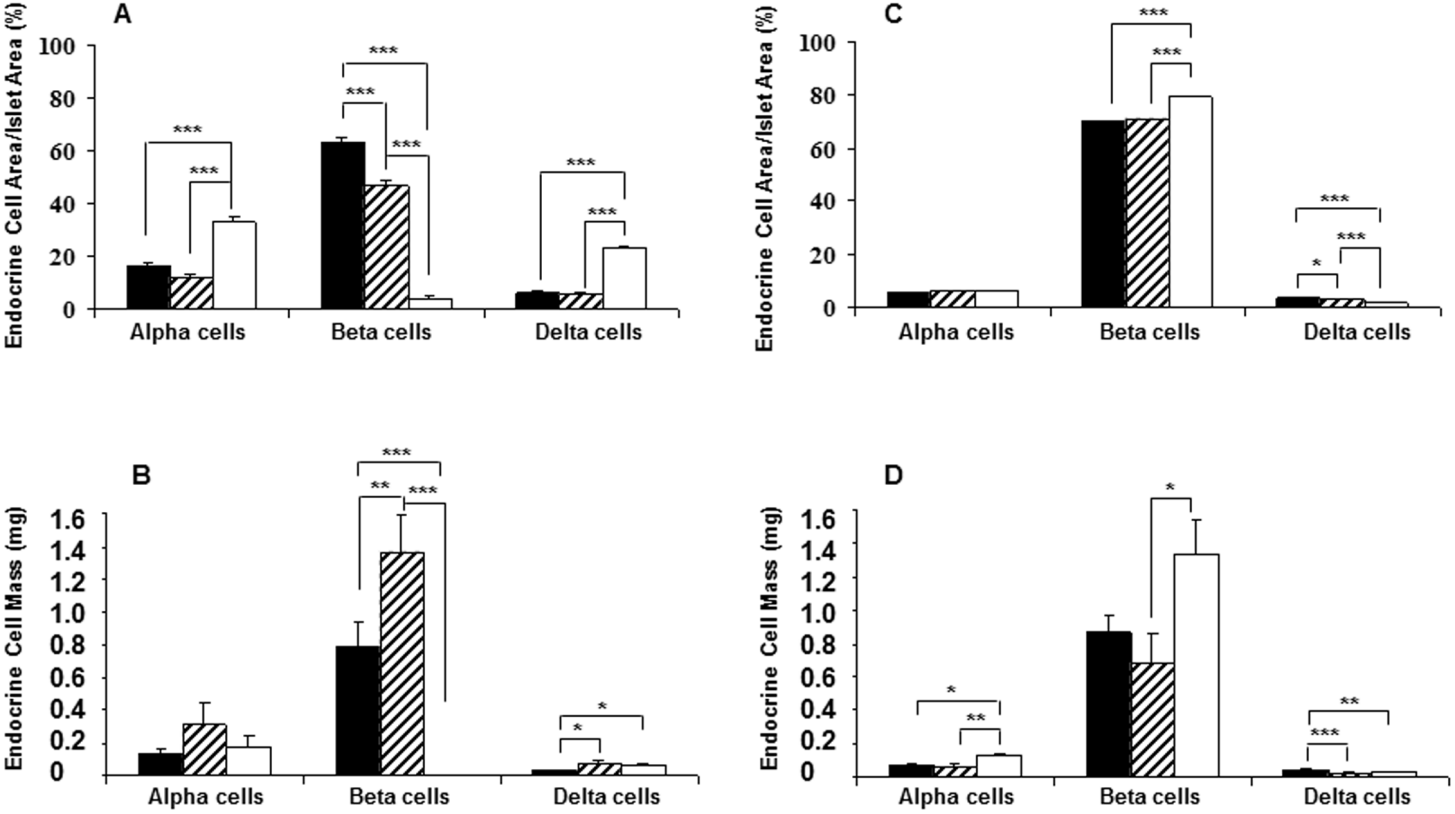
Changes in islet endocrine cell populations during progression of autoimmune diabetes. Alpha, beta and delta-cells were quantified as a percentage of total islet area (A) and total endocrine cell mass (B) in 4-wk old diabetes-prone (black bars), 12-wk old insulitic (hatched bars) and 18–24 wk old diabetic (white bars) female NOD mice. Endocrine cell area (C) and mass (D) was quantified similarly in age-matched female Balb/c mice. Significant changes among groups are indicated as: *p<0.01, **p<0.001 and ***p<0.0001.

**Figure 2:**
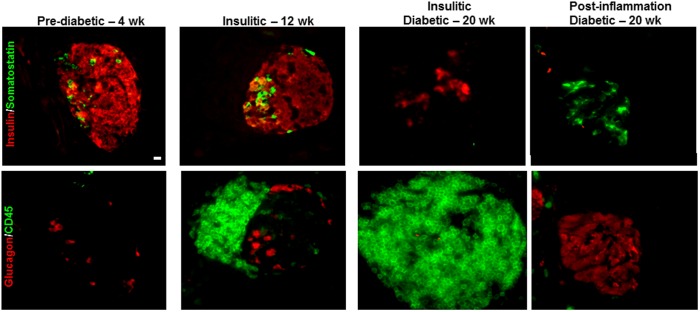
Islet endocrine cell changes during progression of autoimmune diabetes as leukocyte infiltration increases and dissipates. Serial pancreas sections from 4(red) and somatostatin (green), or glucagon (red) and CD45-positive leukocytes (green). Scale bar = 10 µm.

In young (4 wk old) diabetes-prone NOD mice, glucagon-, insulin- and somatostatin-positive cells comprised most of the islet area (84.9±1.8%; Fig. 1a). In older (12 wk old) mice with insulitis and in diabetic (18–24 wk old) NOD mice, the proportion of islet area comprised of these major islet endocrine cell types diminished to 68.0±11.2% and 61.7±4.3%, respectively as the number of infiltrating immune cells in the islets increased markedly, as demonstrated by CD45-staining (Fig. 1 and 2).

As expected, the area comprised of beta-cells as a proportion of total islet area decreased from 63.5±1.4% in the young 4 wk old group to 46.6±2.1% (p<0.05) in the insulitic 12 wk old mice, and was markedly decreased to 4.0±1.1% in the diabetic 18–24 wk old group (Fig. 1a). Accordingly, the total area comprised of beta-cells decreased from 0.51±0.09 mm^2^ in the 4 wk old and 0.53±0.09 mm^2^ in 12 wk old, down to 0.0008±0.0003 mm^2^ in the diabetic 18–24 wk old group (*p<0.001*). This reduction in beta-cell area corresponded to a significant change in pancreatic beta-cell mass among the three groups from 0.8±0.2 mg in the 4 wk old mice to 1.4±0.2 mg in the 12 wk old (*p<0.01*) and 0.002±0.001 mg in the diabetic 18–24 wk old group (*p<0.001*; Fig. 1b). As expected with the loss of beta-cells, the proportion of islet area comprised of glucagon-immunopositive cells increased from 16.4±1.1% in the 4 wk old group to 33.0±1.6% (*p*<0.001; Fig. 1a) in the diabetic group, as individual islets appeared to become increasingly composed of non-beta-cells; however, this was not reflected by an increase in total alpha-cell area (0.09±0.02 mm^2^ in 4 wk and 0.07±0.02 mm^2^ in 20 wk) or alpha-cell mass (0.13±0.03 mg in 4 wk and 0.18±0.06 mg in 20 wk; Fig. 1b). Thus, although many individual islets undergoing beta-cell destruction in the NOD mouse appear to have more alpha-cells, the total alpha-cell mass does not increase as beta-cells are lost. The proportion of islet area comprised of somatostatin-immunopositive cells also increased, from 6.2±1.1% of the islet area in the 4 wk old group to 23.2±1.1% (*p*<0.01) in the diabetic group with delta-cell mass increasing concomitantly as diabetes progressed (0.03±0.01 mg in 4 wk and 0.06±0.01 mg in 20 wk *p<0.05*). These data suggest that islet remodeling occurs as beta-cells are lost in the NOD mouse, with the major proportion of each islet being comprised of beta-cells in young diabetes-prone NOD mice to a mix of alpha and delta-cells in diabetic NOD mice. This was accompanied by a modest increase in delta-cell mass and no change in alpha-cell mass.

**Figure 3:**
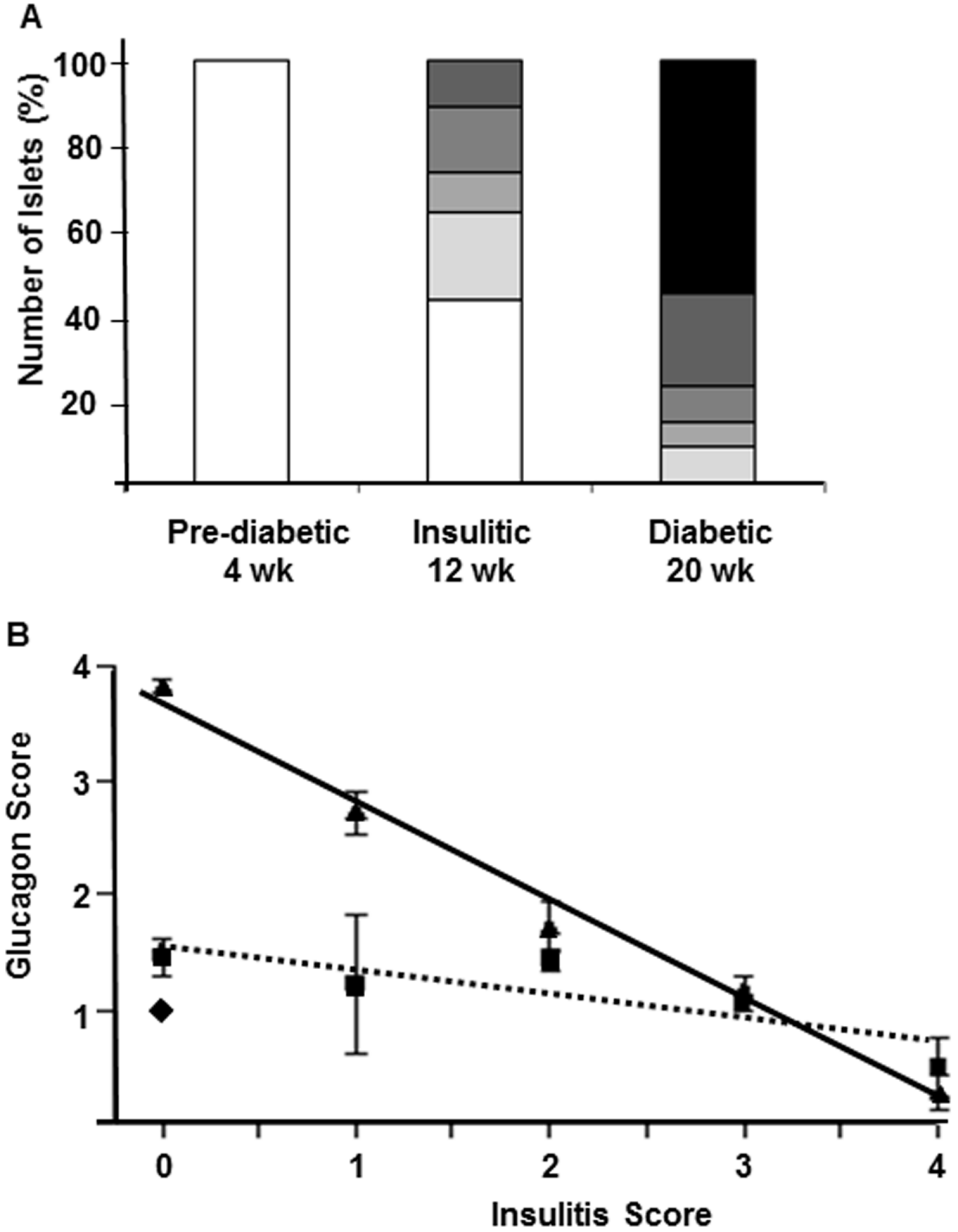
Correlation of insulitis progression with alpha-cell remodeling in individual islets in diabetic NOD mice. Composition of the insulitic lesion in 4-wk old diabetes-prone (islets from 5 mice), 12-wk old insulitic (islets from 7 mice) and 18–24 wk old diabetic (islets from 7 mice) female NOD mice (A, Insulitis score: white = 0 (pre-inflammation), lightest grey = 1 (less than 1/3 of the islet infiltrated), light grey = 2 (between 1/3 and 2/3 of the islet infiltrated), grey = 3 (more than 2/3 of the islet infiltrated), dark grey = 4 (islets with full insulitis) and black = 0 (post-inflammation)). Correlation between the severity of insulitis and degree of glucagon-positive alpha-cells in 4 wk old (diamond), 12 wk old (square) and 20 wk old diabetic NOD mice (triangle; B). Linear regression analysis revealed a negative correlation between the glucagon and insulitis score in the 12 wk old (dotted line R^2^ = 0.79 and p<0.0005) and 20 wk old diabetic (solid line; R^2^ = 0.85 and p<0.0001) groups. Note: islets with an insulitis score of zero may be either pre- or post-inflammation. Islets that were termed “post-inflammation” were easily identifiable; that is, the insulitis had dissipated following destruction of the beta-cells and largely been replaced by alpha and delta-cells.

To address the timing of islet remodeling in correlation to degree of islet inflammation we assessed the severity of insulitis (Fig. 3a) with respect to the distribution of glucagon-positive alpha-cells within each islet in pre-diabetic, insulitic and diabetic NOD mice (Fig. 3b). Both insulitis and distribution of glucagon-positive cells were scored on a scale from zero to four: zero designates no immune or glucagon-positive cells, one designates peripheral location, two and three designate increasing amounts of these cells within the islet and a score of four designates islets with cells evenly distributed throughout the entire islet. It is important to note that islets with an insulitis score of zero may be either pre- or post-inflammation. Islets that were termed “post-inflammation” were easily identifiable; that is, the insulitis had dissipated following destruction of the beta-cells and had largely been repopulated by alpha and delta-cells. As expected, all islets in the pre-diabetic group were found to have no islet inflammation and a peripheral distribution of glucagon-positive cells (Fig. 3b). Islets in the insulitic group demonstrated varying degree of insulitis in half of the islets analyzed and many islets had a peripheral distribution of glucagon-positive cells similar to that seen in the pre-diabetic group. In contrast, islets in diabetic mice with severe hyperglycemia were typically either heavily infiltrated with minimal to no presence of glucagon-positive cells (Fig. 2; insulitic) or exhibited no insulitis but glucagon-positive cells redistributed throughout the core of the islets (Fig. 2; post-inflammation). The infiltrating immune cells were found to precede redistribution of non-beta endocrine cells in islets of diabetic NOD mice as demonstrated by a negative correlation between the mean glucagon and insulitis scores (r^2^ = 0.85; *p*<0.0001; Fig. 3b). Once an islet was completely infiltrated and the beta-cells lost we found little evidence for any of the other three endocrine cell types. Thus, islet cell reorganization appears to commence once the immune cell infiltration dissipates following completed beta-cell degranulation, dedifferentiation or destruction, and may fill part of the void left by the fate of the beta-cells.

Beta-cell mass in age-matched Balb/c mice increased from 0.7±0.2 mg in the 4 wk old group to 1.3±0.2 mg in the 20 wk old group (*p*<0.05; Fig. 1d). We also observed a significant increase in alpha-cell mass from 0.08±0.01 to 0.13±0.02 mg (*p*<0.05; Fig. 1d) between 4 and 20 wk old Balb/c mice. To this end, it is important to note that although alpha-cell mass remained unchanged among the three age groups in NOD mice, the alpha-cell mass at 4 wk was higher in NOD than age-matched Balb/c mice, and was at this young age (0.13±0.03 mg) already similar to levels in 20 wk old Balb/c mice.

We next immunostained sections obtained from the posterior part of the pancreas head for PP-immunopositive cells since we found no increase in the low proportion of this endocrine cell population in the tail of the pancreas of diabetic NOD mice. Compared to 4 wk old NOD and Balb/c mice we found an increase in PP-immunopositive cells in diabetic mice (data not shown). Thus, as for alpha and delta-cells, an increase in the proportion of PP cells also appears to occur during the development of diabetes in the NOD mouse, but is exclusive to those islets in the pancreas head, where PP cells primarily reside.

### Increased Alpha and Delta Endocrine Cell Area and Mass in STZ-Induced Diabetes

**Figure 4:**
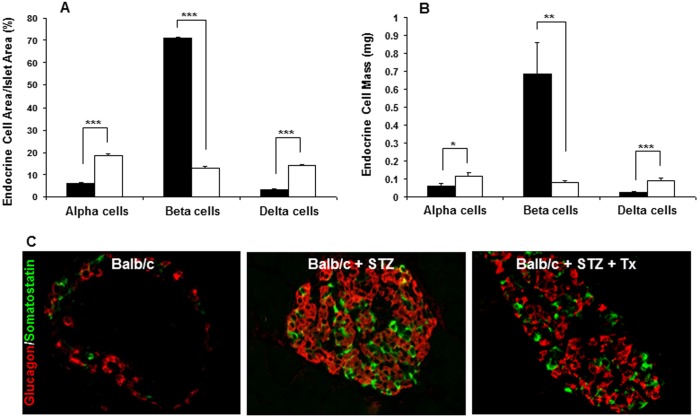
Changes in islet endocrine cell populations in mice with STZ-induced diabetes. Alpha, beta and delta-cells were quantified as a percentage of total islet area (A) and total endocrine cell mass (B) in 12-wk old Balb/c mice (black bars) and 12-wk Balb/c mice with STZ-induced diabetes (white bars) Significant changes among groups are indicated as: *p<0.01, **p<0.001 and ***p<0.0001. Increased proportions of alpha (red) and delta (green) cells coincides with decreased insulin expression (data not shown, C) in 12 wk old female Balb/c mice with STZ-induced diabetes. This was observed irrespective of whether the mice were left untreated (middle panel) or received a syngeneic islet transplantation to restore euglycemia (right panel). Age- and gender-matched Balb/c control islets demonstrated normal endocrine cell distribution with peripherally located alpha and delta-cells and beta-cells throughout the core of the islet (left panel). Scale bar = 10 µm.

We next used a single high-dose of streptozotocin (STZ) to induce beta-cell destruction and acute diabetes with minimal immune infiltration. As expected, immunohistological assessment of pancreatic sections from 12 wk old female Balb/c mice two weeks following STZ treatment revealed markedly increased proportions of alpha- and delta-cells associated with beta-cell loss, similar to the changes in islet endocrine cell populations observed in the NOD mouse model (Fig. 4). Quantitative image analysis demonstrated that the proportion of total islet area comprised of beta-cells in Balb/c mice decreased from 70.8±0.8% in non-treated mice to 13.1±0.5% in STZ-treated mice (*p*<0.0001; Fig. 4a). Beta-cell mass similarly declined with STZ treatment, from 0.68±0.16 to 0.07±0.01 mg (p<0.001; Fig. 4b). The percent of islet area comprised of alpha and delta-cells increased from 6.2±0.4% and 3.4±0.2%, respectively, in non-treated mice to 18.7±0.6% (*p*<0.0001) and 14.1±0.5% (*p*<0.0001) in STZ-treated mice. In contrast to our findings in NOD mice in which alpha-cell mass remained unchanged with onset of autoimmune diabetes, both alpha- (0.06±0.01 vs 0.12±0.02 mg; p<0.01) and delta- (0.025±0.005 to 0.091±0.014 mg; p<0.0001) cell mass increased in STZ-induced diabetes. Since alpha-cell mass increased in the STZ but not the NOD model, the inflammatory milieu in the NOD mouse islet may restrict alpha-cell expansion.

### Insulin Replacement Does Not Prevent Changes in Alpha and Delta-cell Populations in STZ-Induced Diabetes

To determine whether hyperglycemia or hypoinsulinemia may be a stimulus for expansion of non-beta endocrine cells within the islet in diabetes, we restored euglycemia (<11 mM) in STZ-diabetic Balb/c mice by transplantation of 300 syngeneic islets under the kidney capsule. The pancreas of the recipient mice was harvested two weeks post-transplantation and immunostained for endocrine cells. The same increased proportion of alpha and delta-cells throughout the islet was present in the pancreas of islet transplant recipients as observed in both NOD and STZ-induced diabetic mice that did not receive islet transplants (Fig. 4c). By contrast, untreated Balb/c mice displayed normal islet morphology with glucagon- and somatostatin-positive cells residing on the islet periphery.

Recipients of islet transplants were hyperglycemic for 2–3 days following STZ treatment and prior to islet transplantation. To rule out the possible influence of this hyperglycemic period on islet endocrine cell replication, we inserted insulin implants into Balb/c mice immediately following the STZ-induced increase in blood glucose. Within this group, mice were hyperglycemic for no longer than 24 hours, and therefore formed an ideal study group to minimize the possible influence of hyperglycemia. One week after insertion of the insulin implant we immunostained pancreas sections from these mice and observed a similar increased proportion of alpha and delta-cells in the pancreas, and PP cells in the pancreas head, as seen in NOD and STZ-induced diabetic mice (data not shown). No difference in islet morphology was observed between this group and STZ-diabetic mice that did not receive insulin implants. These data suggest that hyperglycemia *per se* is not the only stimulus driving expansion of non-beta endocrine cells, and moreover suggest that the hypoinsulinemia of diabetes is also not essential, since insulin replacement had no impact on islet remodeling in this model. Hence, in STZ-diabetic mice exhibiting no insulitis, expansion of non-beta islet cells occurred regardless of whether insulin was replaced or not.

### Sustained High Levels of Proliferating Beta-cells in Diabetogenic NOD Mice

**Figure 5:**
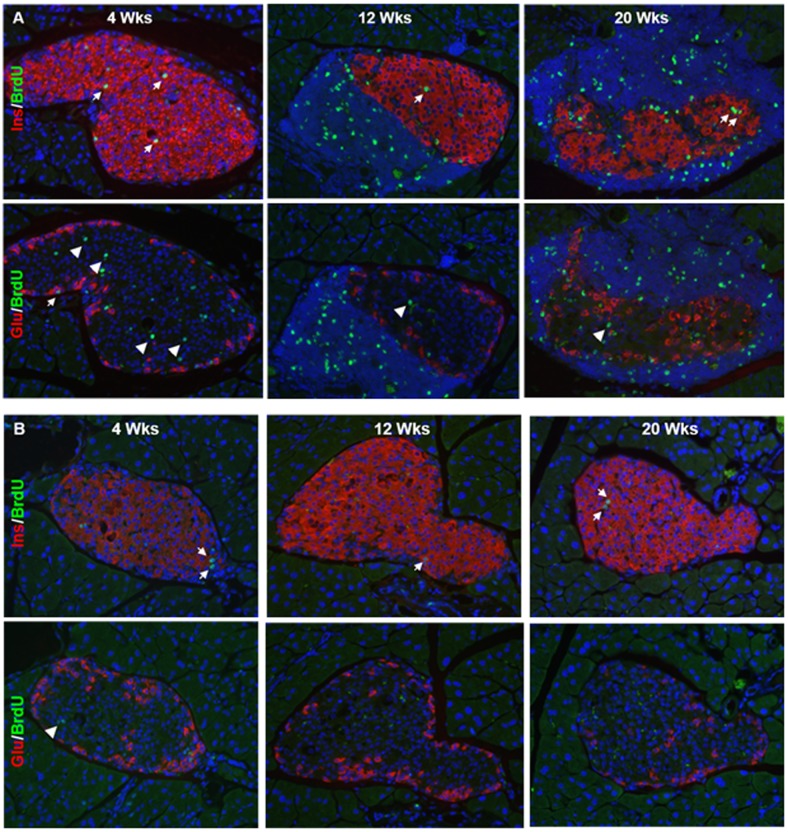
Proliferation of endocrine cells and leukocytes in NOD and Balb/c mouse islets. Co-immunostaining for the nuclear proliferation marker BrdU (green) and islet hormones (red) in 4, 12 and 20 wk old female NOD (A) and age-matched Balb/c mice (B). Sections are counterstained with DAPI to visualize nuclei (and infiltrating immune cells). White arrows indicate double-positive cells, and white arrowheads indicate single BrdU-positive cells within the islet mantel. Scale bar = 10 µm.

**Figure 6:**
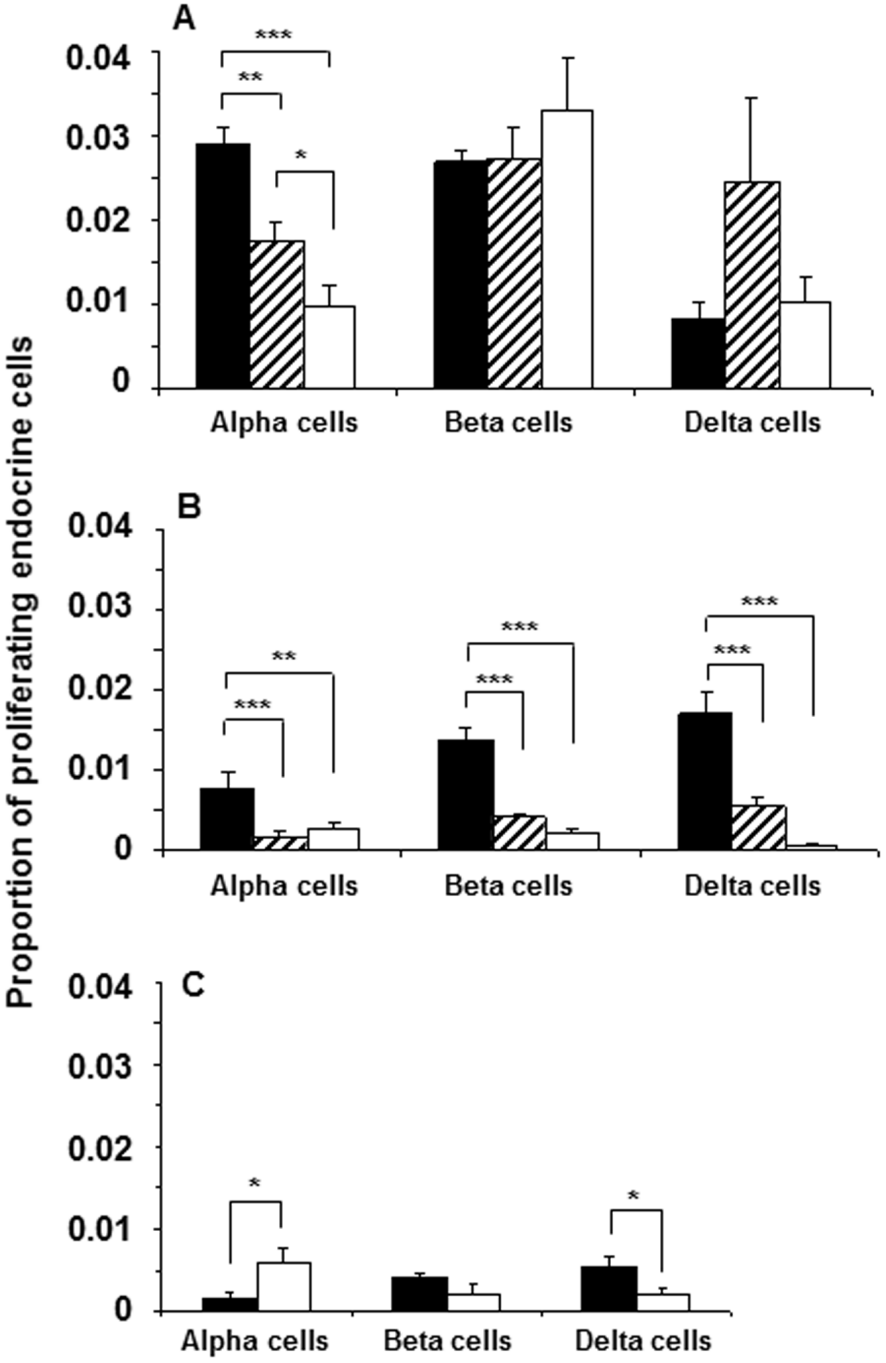
Quantification of proliferating endocrine cells in NOD and Balb/c mice with and without STZ-induced diabetes. Number of proliferating alpha, beta and delta-cells were quantified in relation to total respective endocrine cell number in 4-wk old diabetes-prone (black bars), 12-wk old insulitic (hatched bars) and 18–24 wk old diabetic (white bars) female NOD mice (A). Number of proliferating endocrine cells was quantified similarly in age-matched female Balb/c mice (B), and in 12 wk old Balb/c mice without (black bars) or with (white bars) STZ-induced diabetes (C). Significant changes among groups are indicated as: *p<0.01, **p<0.001 and ***p<0.0001.

To determine the degree of proliferating endocrine cells during ongoing beta-cell destruction in the NOD mouse, we quantified BrdU-labeled pancreas sections immunostained for glucagon, insulin or somatostatin (Fig. 5a and 6a). We observed sustained high levels of proliferating insulin-positive cells in all age groups (4 wk: 2.6±0.1%; 12 wk: 2.7±0.3%; 20 wk: 3.3±0.5%). By contrast, the high level of proliferating glucagon-positive cells found in young NOD mice (2.9±0.2%) decreased to 1.7±0.2% (*p*<0.01) in 12 wk old mice and further to 0.9±0.2% (*p*<0.001) in 20 wk old mice. Age-matched Balb/c mice demonstrated a similar significant decrease over time in the number of proliferating alpha, beta and delta-cells although the overall proliferative rate was ∼3-fold lower in this strain (Fig. 5b and 6b) compared to NOD mice. In the STZ model of diabetes, we found a significant increase in the number of proliferating alpha-cells compared to diabetic NOD mice (Fig. 6c) correlating with the significant increase in alpha-cell mass observed in this model (Fig. 4b). The degree of proliferation was confirmed by double immunostaining endocrine cells with Ki67 as an alternative marker (Figure S1). Both our pre-diabetic NOD mice and STZ-diabetic mice that received islet transplants to normalize fasting glycemia were found to have impaired glucose tolerance (data not shown). The higher post-prandial glucose excursions in these animals could contribute to islet cell proliferation despite the presence of normal fasting glycemia.

In 12 wk old NOD mice, neighboring islets within the same pancreatic section often demonstrated strikingly different proportions of islet endocrine cells. Thus, while many islets in this pre-diabetic age group showed, as expected, little or no immune infiltration and normal islet endocrine cell morphology, those islets with progressive insulitis and clear beta-cell loss typically demonstrated increased numbers of BrdU-positive non-beta endocrine cells. In contrast, in 20 wk old diabetic NOD mice with clear islet pathology and few existing insulin-positive beta-cells, we observed a marked proliferation of insulin-positive cells (Fig. 5a and 6a). Thus, islet endocrine cell replication appears to be initiated prior to the development of overt diabetes, is not specific for non-beta islet cells, and is more closely associated with beta-cell loss rather than with hyperglycemia *per se* in this model.

### Expression of Other Beta-cell Markers in Heavily Infiltrated Islets of Diabetic NOD Mice

**Figure 7:**
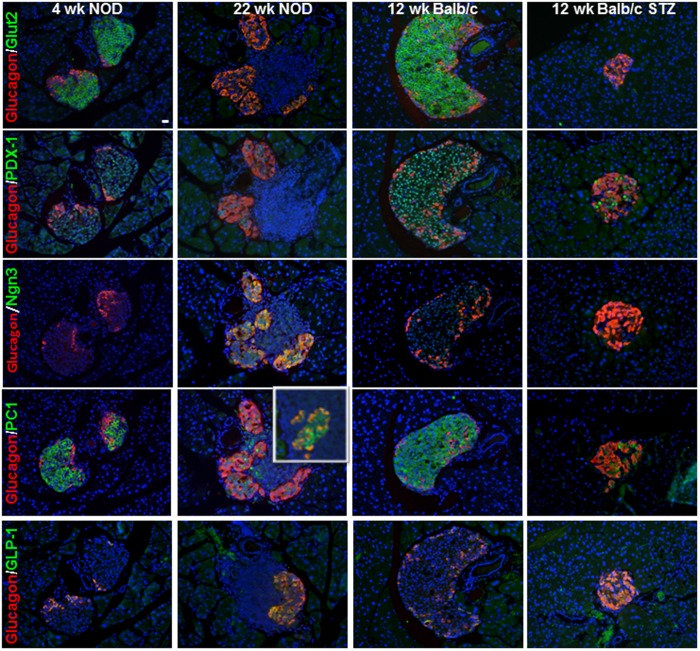
Identification of beta-cell markers in islets from diabetic NOD mice compared to STZ-induced diabetic Balb/c mice. Representative pancreas sections from 4/c mice without or with STZ-induced diabetes were immunostained for glucagon (red) and Glut-2, PDX-1, Ngn3, PC-1/3 and GLP-1 (green, as indicated). Sections are counterstained with DAPI to visualize nuclei (and infiltrating immune cells). The insert on the glucagon and PC-1/3 panel indicates double-positive cells in an area where all glucagon cells are positive for PC-1. Scale bar = 10 µm.

Differentiation and potential replication of both pan-endocrine progenitor cells and non-beta islet endocrine cells during the development of diabetes may occur as a possible mechanism to either increase the pool of beta-cell progenitors to replenish destroyed beta-cells or to facilitate the ensuing islet remodeling. To address whether insulin-negative cells in the core of heavily infiltrated NOD islets and STZ-diabetic mice may be degranulated, dedifferentiated [8] or quiescent beta-cells [21], we stained pancreas sections for three additional beta-cell markers: the transmembrane glucose transporter 2 (GLUT2), the transcription factor pancreatic and duodenal homeobox 1 (PDX-1) as well as the fetal and neonatal transcription factor Neurogenin 3 (Ngn3). As expected, both GLUT2 and PDX-1 were expressed in the islet core in 4 wk old prediabetic NOD and 12 wk Balb/c mice, whereas these markers were not observed in heavily infiltrated islets in diabetic NOD mice and GLUT2 was altogether missing from diabetic islets (Fig. 7). Ngn3-positive cells that gives rise to alpha, beta, delta and PP cells is expressed during embryogenesis in early endocrine cells by E8.5, peak by E15.5 and decline after birth [22], [23], [24]. Ngn3-immunopositive cells were clearly present in neonatal day zero NOD and Balb/c control mouse islets (data not shown). After this time point Ngn3 was not detectable in control mice; however, in diabetic NOD mouse islets undergoing remodeling we found Ngn3-expressing cells usually colocalized with glucagon, whereas no Ngn3-positive cells were detected in the islets of Balb/c mice with STZ-induced diabetes (Fig. 7). These data raise the possibility that quiescent beta-cells in diabetic NOD mice revert to expressing Ngn3 and that this may be part of an islet remodeling process in diabetic NOD mice.

Immunoreactivity for the prohormone convertase PC1/3 was found in the islet cores as well as in some peripherally-located alpha-cells in both young diabetes-prone NOD and in Balb/c mice (Fig. 7). In diabetic mice, many PC1/3-immunopositive cells were also positive for glucagon; these cells tended to reside in remodeled areas of islet in NOD mice. In the STZ model PC1/3-immunopositive cells were scattered throughout the islet and were either glucagon or insulin double-positive. PC1/3 is preferentially expressed in beta-cells and cleaves proinsulin to insulin but is typically absent in alpha-cells. When PC1/3 is expressed in alpha-cells it allows for alternative processing of proglucagon to produce glucagon-like peptide 1 (GLP-1), in addition to glucagon that is processed from the same prohormone by PC2 [25], [26]. In support of these findings, we found GLP-1 co-localizing with many alpha-cells in both the NOD and STZ models of diabetes (Fig. 7).

### Serum hormone levels reflect changes in endocrine cell mass during diabetogenesis

**Figure 8:**
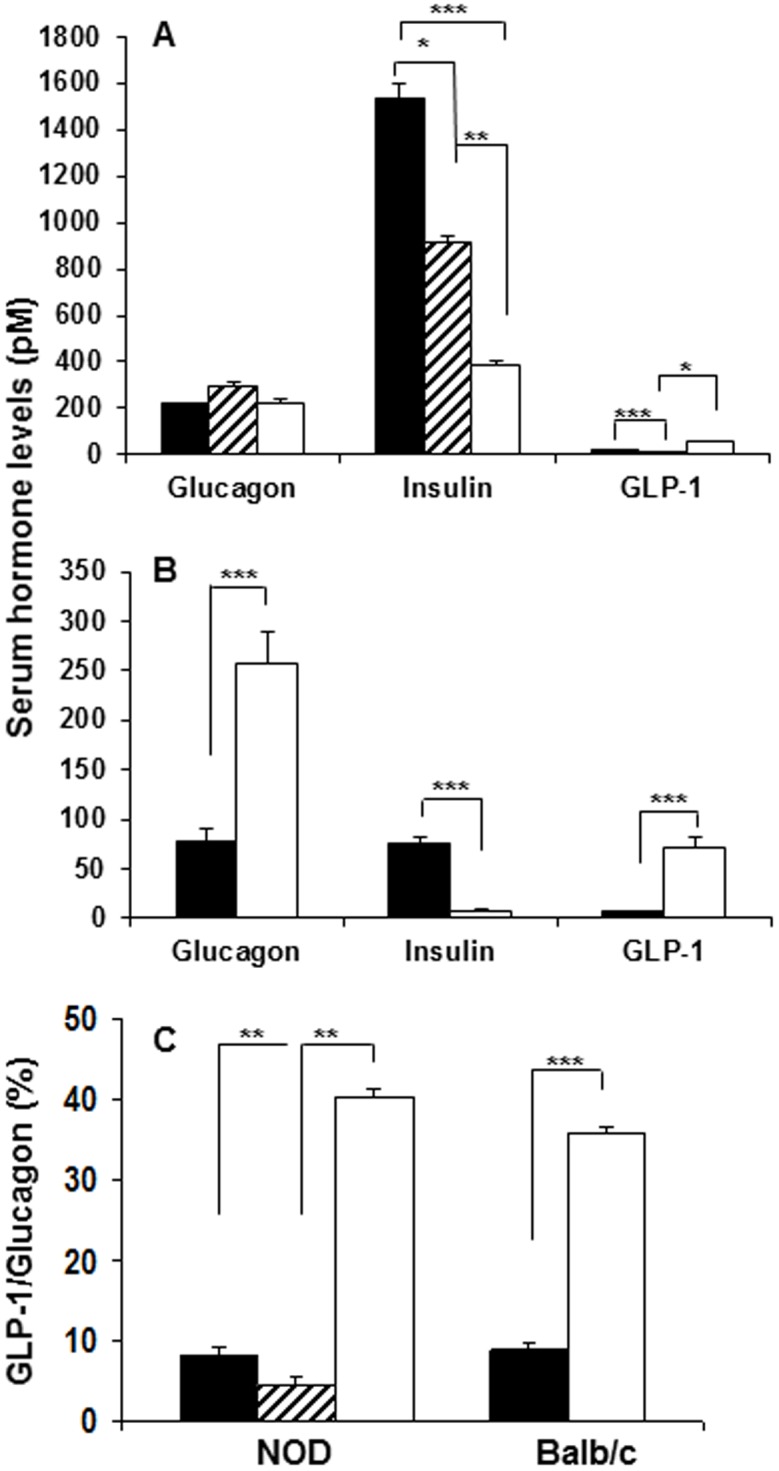
Serum hormone levels reflect changes in endocrine cell mass. Levels of glucagon, insulin and GLP-1 in serum from 4-wk old diabetes-prone (black bars), 12-wk old insulitic (hatched bars) and 18–24 wk old diabetic (white bars) female NOD mice (A), and in 12 wk old Balb/c mice without (black bars) or with (white bars) STZ-induced diabetes (B). The ratios of the proglucagon gene products in percent is given for both the NOD and Balb/c mice (C). Significant changes among groups are indicated as: *p<0.01, **p<0.001 and ***p<0.0001.

As expected, levels of circulating insulin decreased in both the NOD and STZ model of diabetes (Fig. 8a and 8b). By contrast, levels of circulating glucagon remained constant in NOD mice as the disease progressed, in accordance with our finding that alpha-cell mass does not increase as beta-cells are lost in this mouse model of diabetes (Fig. 1b). In STZ-diabetic mice, in which alpha-cell mass was increased, serum glucagon levels were elevated 3-fold over controls (Fig. 4). Interestingly, serum GLP-1 levels were elevated in both models of diabetes (3-fold for the NOD and 12-fold for the STZ model), likely associated with the increased expression of PC1/3 in the glucagon-positive cells in diabetic mice. The increased GLP-1 in diabetic animals resulted in a significant change in the ratio of the proglucagon gene products in support of GLP-1 (Fig. 8c). The substantially higher GLP-1 levels in the STZ model could be due to the increased alpha-cell mass in addition to alpha-cell PC1/3 expression.

## Discussion

We examined the reorganization of islet endocrine cell populations during the development of diabetes in the NOD and STZ mouse models of diabetes. As expected, with loss of beta-cells, an increase in the relative proportion of alpha and delta-cells was observed in both models of diabetes. This increase in the proportion of non-beta endocrine cells in diabetic islets appeared to be associated not only with beta-cell-specific loss but with proliferation of all islet endocrine cell types, including beta-cells, and in NOD mice begins well before the development of overt hyperglycemia. We could not ascribe the stimulus for the increased proliferation of islet endocrine cells during the development of diabetes to the presence of infiltrating immune cells, since this phenomenon was also observed in STZ-induced diabetic islets in which inflammation was not observed. An important difference between the NOD and STZ models of diabetes is our finding that alpha-cell proliferation and mass only increases following STZ-induced beta-cell destruction, but not following autoimmune beta-cell loss in the NOD model. These differences raise the possibility that the inflammatory milieu in NOD mouse islets may restrict alpha-cell expansion. To this end, various alternative NOD mouse models of either accelerated, delayed or inhibited autoimmunity could be explored in order to assess changes in alpha cell mass due to the altered or decreased degree of insulitis [27], [28], [29]. The presence of hyperglycemia or hypoinsulinemia did not seem to explain the difference in islet remodeling between the two models given that insulin replacement by insulin implant or islet transplantation in STZ-diabetic mice did not prevent alpha-cell expansion. The changes in endocrine cell mass were reflected in serum hormone levels; hence, while insulin levels decreased in both models of diabetes, glucagon levels increased only in the STZ model of diabetes. Notably, GLP-1 levels increased in both the NOD and STZ models of diabetes, associated with PC1/3 and GLP-1 expression in glucagon-producing cells. Another notable difference identified was the expression of Ngn3 following beta-cell loss only in diabetic NOD mice and Ngn3 expression at this stage was found to be primarily cytoplasmic and colocalized with glucagon in heavily infiltrated islets.

Dynamic changes in beta-cell mass have been reported as a response to increased metabolic demand, for example during pregnancy or insulin resistance, or following pancreatectomy and beta-cell destruction. A previous report noted proliferation of beta-cells soon after onset of insulitis as a compensatory mechanism to replenish insulin levels during active beta-cell destruction in 8 week old NOD mice [30]. Another study used flow cytometry to show that elevated blood glucose is the driving force behind beta-cell proliferation during diabetogenesis, whereas non-beta endocrine cells do not show a similar glucose-dependent proliferative response [31]. Our finding of BrdU- and Ki-67 positive alpha, beta, delta, and PP cells in all three age groups of NOD mice suggests that islet cell proliferation accompanying beta-cell death is likely an important contributor to the changes in islet morphology in diabetic mice. Once beta-cell proliferation has been outstripped by beta-cell death (or perhaps dedifferentiation), an increased proportion of non-beta islet endocrine cells appear in the islet core, filling the void left by loss of beta-cells. However, in contrast to a previous report of increased numbers of PP cells in the tail of the pancreas in NOD mice, we observed PP cells to be increased exclusively in the head of the pancreas in 12 and 20 wk old NOD mice [5]. In non-diabetic Balb/c mice we found a general decrease in cell proliferation with age (Fig. 7b) confirming previously reported findings [32], [33].

Using an inducible autoantigen-specific model to generate experimental autoimmune diabetes, as well as the NOD model, Pechhold et al demonstrated that islets isolated from newly diabetic mice showed a significant loss of not only beta-cells, but also alpha-cells [6]. These findings may seem contradictory to our data demonstrating sustained alpha-cell mass throughout NOD mouse diabetogenesis; however an important difference was their use of newly diabetic mice, whereas mice in our diabetic cohort were studied after two weeks of hyperglycemia. In our group of diabetic mice with sustained hyperglycemia, the majority of islets were either heavily infiltrated with minimal to no glucagon-positive cells (similar to Pechhold et al), or exhibited no insulitis with glucagon-positive cells distributed throughout the core of the islets suggesting that alpha-cell mass may be dependent on the stage of disease.

Several studies have addressed whether beta-cells lost during the development of diabetes may be replenished by potential islet cell progenitors such as duct cells [34], acinar cells [15], trans-differentiation of non-beta islet endocrine cells [10], by replication and regeneration of existing beta-cells [12], [13], [35] or as recently proposed dedifferentiation into progenitor-like cells expressing early transcription factors such as Ngn3 and Oct4 [8]. As reported previously [9], we occasionally observed glucagon and insulin double-positive cells in both 12 and 20 wk old NOD mice, although we have no evidence that these double-positive cells are progenitors or trans-differentiating cells, information best obtained from lineage tracing studies [11].

An important novel finding in our studies was the expression of the Notch target gene Ngn3 in islets from diabetic NOD mice but not those of Balb/c mice with STZ-induced diabetes. This transcription factor is primarily expressed during embryogenesis and gives rise to endocrine progenitors that ultimately differentiate into alpha, beta, delta and PP cells [22], [23]. Ngn3 is down-regulated following prenatal development and typically not expressed in adolescent or adult islets; however, expression of Ngn3 in adult islet tissue has been described in models of beta-cell dedifferentiation or diabetes [8], [36]. To our knowledge this is the first study demonstrating expression of Ngn3 in diabetic NOD islets. Given that the majority of Ngn3-positive cells also immunostained positive for glucagon, we speculate that these cells could be beta-cells undergoing dedifferentiation.

Infiltrating immune cells may secrete cytokines and growth factors that could stimulate endocrine cell replication and have been proposed to contribute to islet neogenesis during prolonged hyperglycemia [37]. This notion is further supported by a study indicating that the presence of islet inflammatory cells is essential for increased beta-cell proliferation [38]. To test whether immune cell stimulus is necessary for diabetic islet cell proliferation we used a diabetes model with minimal immune infiltration, administration of a single high-dose STZ, and observed similar increased alpha- and delta-cell mass in these islets despite absence of any noticeable immune infiltration. Indeed, redistribution of the alpha and delta-cell populations in individual islets in the NOD mouse appeared greatest after the insulitis had dissipated, suggesting that the presence of infiltrating immune cells may impede expansion of the non-beta endocrine cell population in the diabetic islet. Our findings do not rule out that immune cells may contribute to stimulation of non-beta and perhaps beta-cell regeneration in autoimmune diabetes. Both splenocytes [39], [40], [41], [42] and transplanted bone marrow cells [43] have been shown to home to the pancreas in NOD mice or following induction of diabetes with multiple low-dose STZ treatment and may contribute to islet cell regeneration in these models.

When STZ-diabetic mice received insulin implants or syngeneic islet transplants, euglycemia was restored within 24 hours following STZ treatment. These mice experiencing minimal hyperglycemia nevertheless displayed very similar islet morphology, including increased proportions of islet alpha and delta-cells, to that seen in the untreated STZ-diabetic animals. Other groups have demonstrated that short-term or mild sustained hyperglycemia induces an increase in beta-cell replication in rodents [44], [45]. Our findings indicate that other mechanisms besides hyperglycemia or lack of insulin also may stimulate islet cell remodeling in diabetic animals. Indeed, as reported by others [46], we found elevated expression of PC1/3 in islet alpha-cells of diabetic mice associated with increased circulating levels of GLP-1, a stimulator of beta-cell proliferation. An influence of elevated blood glucose on islet cell proliferation in our studies cannot be ruled out however, given the short period of hyperglycemia experienced even by mice that received an immediate insulin implant immediately following STZ treatment.

In summary, alpha-cell mass is increased following STZ-induced beta-cell destruction in Balb/c mice, but not following autoimmune beta-cell loss in NOD mice. In STZ-diabetic mice exhibiting no insulitis, islet remodeling of non-beta endocrine cells occurred regardless of whether insulin was replaced or not. We suggest that mechanisms other than hyperglycemia and the presence of infiltrating immune cells are essential for non-beta islet endocrine cell remodeling in diabetic NOD mice and lead us to suggest that these cells may redistribute to fill the void left by loss of beta-cells within the diabetic islet once insulitis dissipates. These data point to important differences in the STZ and NOD models of diabetes and suggest that an increased understanding of the local factors regulating islet cell proliferation may lead to new strategies to maintain normal islet architecture in diabetes.

## Supporting Information

Figure S1 **Ki-67 staining of proliferating endocrine cells compliment BrdU staining.** Co-immunostaining for the nuclear proliferation marker Ki-67 (red) and islet hormones (green) in 12 wk old female NOD and Balb/c mice. Multiple Ki-67 (green) and CD45-positive leukocytes (red) were present in 12-wk old insulitic NOD mice (upper right panel). Scale bar = 10 µm.
(TIF)
