# Supplementary figures and images for: Islet Remodeling in Female Mice with Spontaneous Autoimmune and Streptozotocin-Induced Diabetes
Source: PLoS One. 2014 Aug 7;9(8):e102843. doi: 10.1371/journal.pone.0102843 (PMC4125302; doi:10.1371/journal.pone.0102843)

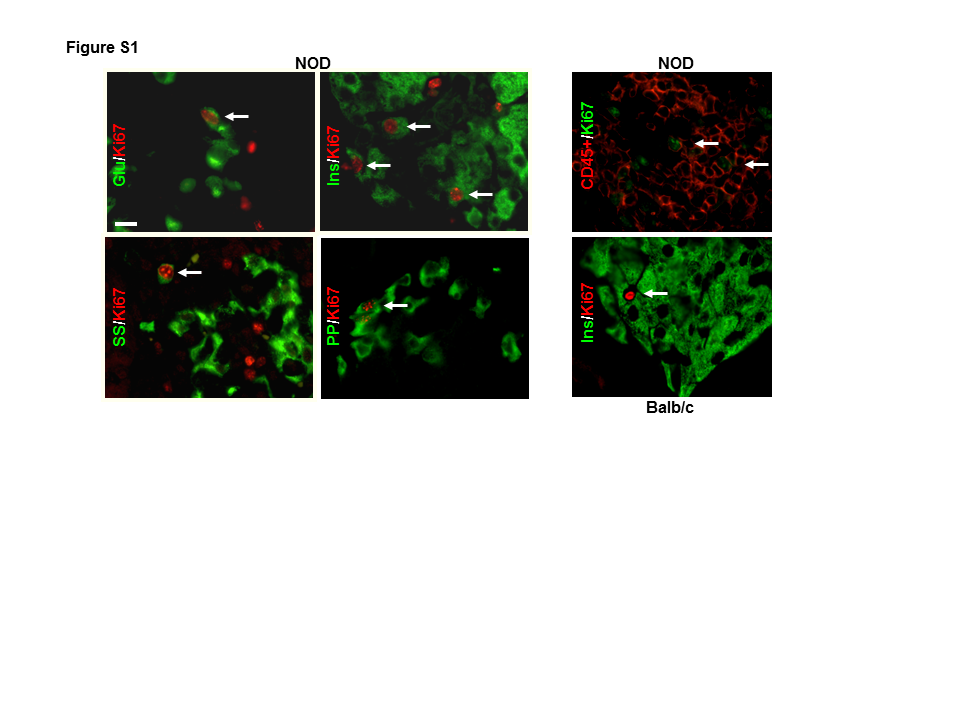

Supplement: Figure S1 — Ki-67 staining of proliferating endocrine cells compliment BrdU staining. Co-immunostaining for the nuclear proliferation marker Ki-67 (red) and islet hormones (green) in 12 wk old female NOD and Balb/c mice. Multiple Ki-67 (green) and CD45-positive leukocytes (red) were present in 12-wk old insulitic NOD mice (upper right panel). Scale bar = 10 µm. (TIF) [file pone.0102843.s001.tif]
